# Recent Advances in Three-Dimensional Multicellular Spheroid Culture and Future Development

**DOI:** 10.3390/mi12010096

**Published:** 2021-01-18

**Authors:** Honglin Shen, Shuxiang Cai, Chuanxiang Wu, Wenguang Yang, Haibo Yu, Lianqing Liu

**Affiliations:** 1 School of Electromechanical and Automotive Engineering, Yantai University, Yantai 264005, China; shl15506583573@163.com (H.S.); caisx8411@ytu.edu.cn (S.C.); wuchuanxiang0724@163.com (C.W.); 2 State Key Laboratory of Robotics, Shenyang Institute of Automation, Chinese Academy of Sciences, Shenyang 110016, China; yuhaibo@sia.cn (H.Y.); lqliu@sia.cn (L.L.)

**Keywords:** cell spheroids, three-dimensional cell culture, tissue engineering

## Abstract

Three-dimensional multicellular spheroids (MCSs) have received extensive attention in the field of biomedicine due to their ability to simulate the structure and function of tissues in vivo more accurately than traditional in vitro two-dimensional models and to simulate cell–cell and cell extracellular matrix (ECM) interactions. It has become an important in vitro three-dimensional model for tumor research, high-throughput drug screening, tissue engineering, and basic biology research. In the review, we first summarize methods for MCSs generation and their respective advantages and disadvantages and highlight the advances of hydrogel and microfluidic systems in the generation of spheroids. Then, we look at the application of MCSs in cancer research and other aspects. Finally, we discuss the development direction and prospects of MCSs

## 1. Introduction

**Figure 1:**
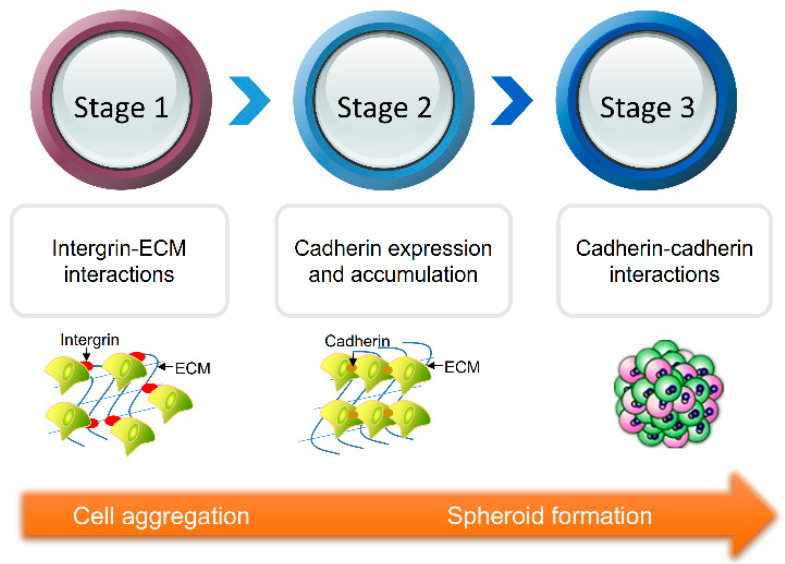
The multicellular spheroid (MCS) formation process is divided into three stages. In the first stage, the extracellular matrix (ECM) acts as a long link head, and the scattered single cells form loose aggregates under the action of integrins. In the second stage, the epithelial cadherin accumulates, and the aggregates enter the delayed phase of suspension of compaction. In the third stage, the loose cell aggregates form dense spheroids under the strong hemophilic interaction of epithelial cadherin.

In the field of biomedicine, cell biology is researched by culturing cells in vitro. Traditionally, cells are cultured in a petri dish or a culture bottle using a two-dimensional culture method. In 1943, Earle created a monolayer cell culture method. To a certain extent, a traditional two-dimensional cell culture method is simple to operate and helps to better the growth, proliferation, and differentiation of cells in the body. However, it cannot simulate the complex biological microenvironment in the body and lose tissue-specific properties, which results in a certain discrepancy between the obtained experimental results and the situation in the body. In the 1980s, Weaver systematically summarized the relationship between cells and the extracellular matrix (ECM) and constructed a three-dimensional cell culture (TDCC) model in the study of breast cancer cells, which gave birth to three-dimensional culture technology. Three-dimensional culture can better simulate the cell–cell and cell–extracellular matrix interactions in vivo, and provide a more realistic microenvironment for the cell culture in vivo. In the three-dimensional cell culture, cells spontaneously aggregate and form compact multicellular spheroids (MCSs) when combined with cadherin [1]. Specifically, a three-dimensional cell culture process is divided into three stages (Figure 1). In the first stage, the ECM with multiple RGD motifs acts as a long link head, and the scattered single cells form loose aggregates under the action of integrins. In the second stage, the epithelial cadherin expresses and accumulates, and the aggregates enter the delayed phase of suspension of compaction. In the third stage, the loose cell aggregates form dense spheroids under the strong hemophilic interaction of epithelial cadherin by forming cadherin-cadherin binding [2]. Furthermore, extracellular mechanical cues were transduced to actin filaments by integrin of cells, which is an essential process for spheroid self-assembly. MCSs have received extensive attention as an important three-dimensional model for cancer research [3,4,5], anti-cancer drug screening [6,7], drug toxicity analysis [8], and tissue engineering [9,10,11]. For example, in the field of drug screening, it is necessary to evaluate the efficacy of in vitro tumor models before conducting animal experiments and clinical experiments. Multicellular tumor spheroids (MCTCs) are used as avascular tumor models for anti-cancer drug screening due to their metabolic and proliferation gradient distribution, similar to in vivo tumor tissues [3]. The application of MCSs helps to reduce the cost and ethical/legal concerns of using animals for experiments in laboratories, while helping to build a bridge between in vivo and in vitro biology research. So far, a series of cells have been explored for the production of MCSs, including cancer cells [4,12], induced pluripotent stem cells [13,14], and fibroblasts [15,16]. Although MCSs models have been widely recognized in the field of biomedicine, their development and application still suffer from limitations when it comes to how to achieve high-throughput generation, reduce the cost and difficulty of generation, and further improve the accuracy of the biochemical signals provided by the spheroid generation process. Although MCSs generation methods have been reviewed by other researchers [17,18,19], comprehensive and systematic reviews are still rare on the development and application of these methods. Therefore, this article reviews recent advances in MCSs research. First, we present a series of MCSs generation methods. Then, we cover their working principles and give examples of their real-world applications. The materials used in the generation of MCSs are summarized. Second, the advantages and disadvantages of various methods are analyzed. Then, the application fields of MCSs are summarized. Finally, the current situation and future development directions of MCSs are discussed.

## 2. Methods for MCSs Generation

MCSs were originally made by A. Moscona and H. Moscona through the self-assembly of cell suspensions. They found that independent limb-bud cells and mesonephric cells of early chick embryos could reconstruct tissue-like connections in vivo and restore their unique histotypical development [20]. Generation efficiency, convenience, economy, difficulty in forming spheroids, and size consistency of MCSs are used as indicators for evaluating spheroid generation methods. Although traditional MCSs preparation methods are exemplified by non-adhesive surface liquid covering, the microwell arrays method, hanging drop, rotating flask, and external force method have been widely used; they generally deliver low production efficiency, require a large amount of labor, and have difficulty in controlling the spheroid size. Despite some innovations on these methods, their inherent disadvantages hinder their further development in the field of biomedicine. With the development and progress of some technologies such as micro/nanofabrication, cell imaging and optics, new MCSs generation technologies have been developed, among which microfluidic technology and cell scaffold technology are typical examples. These technologies can not only enable high-throughput preparation of MCSs, but also provide a deeper understanding of the formation process of MCSs and the mechanism of intercellular interactions. Moreover, researchers use hydrogels to simulate the ECM during cell growth, thus providing more realistic in vivo microenvironment for cells. Table 1 is a summary of various MCSs generation methods and their advantages and disadvantages.

### 2.1. Traditional Generation Methods

#### 2.1.1. Non-Adhesive Surface Liquid Covering (the Microwell Arrays Method)

**Figure 2:**
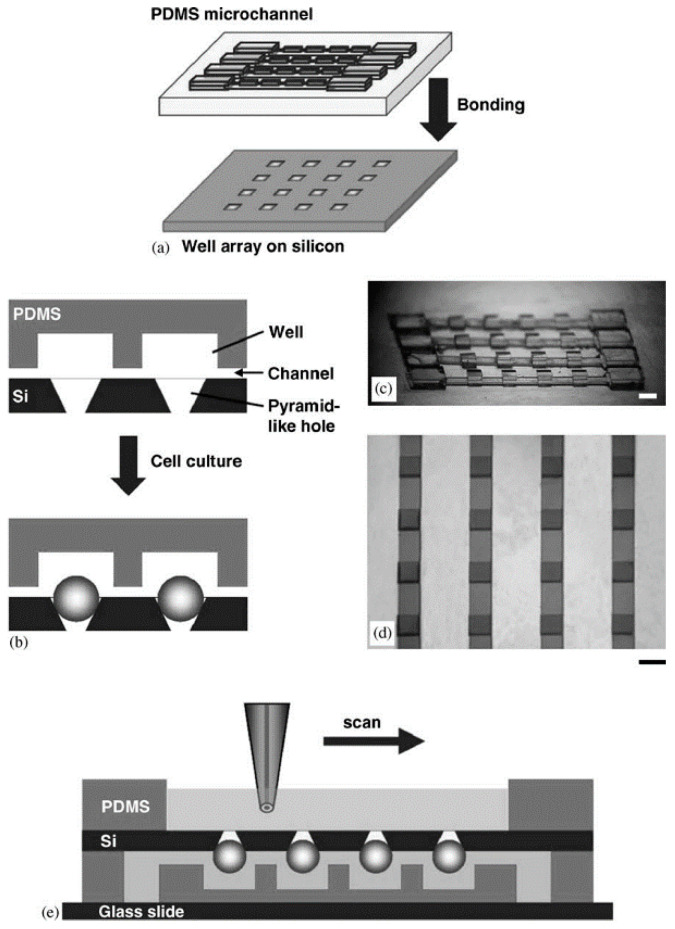
A schematic illustration of the concept (**a**) and cross-section (**b**) of the spheroid chip. Polydimethylsiloxane (PDMS) microchannels and the array of wells were bound to a silicon substrate. (**c**) The master mold for fabricating PDMS microchannels using two-step photolithography. (**d**) The fabricated microchannels which were attached to the glass substrate. (**e**) The tip scanned over the spheroid for measurement. The scale bar is set at 1 mm. (Reproduced from Reference [23]).

By injecting the cell suspension into a culture vessel with a non-adhesive surface, the cells are prevented from adhering to the wall. Slightly shaking the vessel and stirring the solution promotes the aggregation of cells in the suspension solution and finally forms a spheroid. The non-adhesive surface can be prepared by a thin coating of agarose and agar, or it can be coated with polymers such as non-sticky poly-hydroxyethyl methacrylate (HEMA) and poly-2-hydroxyethyl methacrylate (PHEMA) [21]. Metzger et al. used the liquid covering method to prepare single and co-cultured spheroids composed of human osteoblasts (HOB), normal human dermal fibroblasts (NHDF), and human dermal microvascular endothelial cells (HDMEC) [22]. The spheroids formed by simple suspension culture are irregular, also with poor preparation efficiency, inconvenience to collect them, and the inability to monitor how they are formed. Combining microwell arrays and a suspension culture can achieve high-throughput preparation of spheroids. Polydimethylsiloxane (PDMS) was fabricated by photolithography, and the cell viability of the spheroids was monitored by scanning electrochemical microscopy (SECM) based on noninvasive measurement [23] (Figure 2). The width of the microchannel was 700 μm and the height of the microchannel was 100 μm. The size of the cell spheroids depended on the concentration of the injected cell suspension. Furthermore, the respiratory activity of the spheroid was evaluated using SECM. The spheroid chip was placed on a glass slide with the channel side at the bottom, firstly. Then, the PDMS well was placed on the side with the smaller opening of the chip. After adding the HEPES-based saline solution and placing the microelectrode and reference electrode in the well, the tip was scanned above the chip surface to detect the oxygen concentration. The microwell arrays method has been widely used in the preparation of spheroids [7,24,25,26,27,28].

#### 2.1.2. Hanging Drop

**Figure 3:**
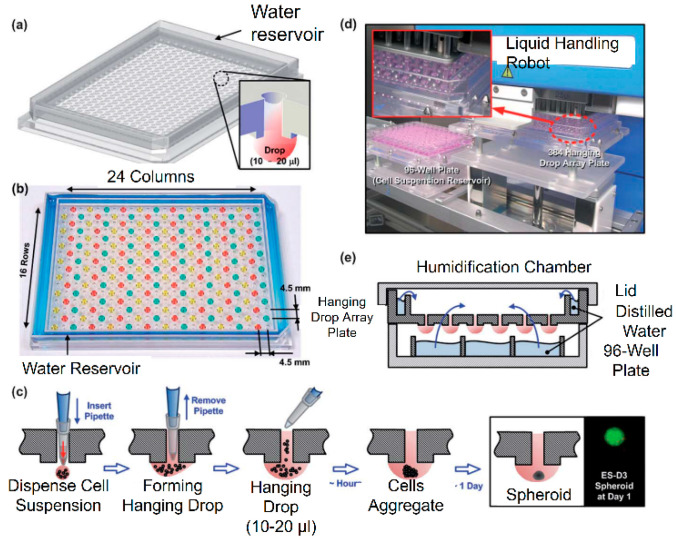
(**a**) The 384 hanging drop spheroid culture array plate designed by Tung. (**b**) The photo of the array plate. (**c**) The hanging drop formation process in the array plate. (**d**) The arrays plate operated by liquid handling robot. (**e**) The array plates were cultured in a humidification chamber. (Reproduced from Reference [32]).

In the traditional hanging drop method, cells are injected into the culture medium to form a suspension. The cell suspension is dropped onto the bottom plate of the culture plate through a dropper, and then the bottom plate is turned upside down. The droplets hang upside down on the bottom plate under the action of surface tension. Under the action of gravity, it gathers at the bottom of the droplet to form a spheroid [29]. Upreti et al. used the hanging drop method to culture green fluorescent protein (GFP)-4T1 cells and 2H11 mouse endothelial cells, forming tumor-cell-only and tumor–endothelia cell spheroids [30]. As the concentration of the cell suspension increases, the volume of the spheroid becomes larger. Cancer cells and stromal cells are co-cultured in collagen gel to form a multicellular heterospheroid tumor model. Compared with the two-dimensional model, the three-dimensional heterospheroid model is more resistant to doxorubicin [31]. To reduce the influence of the inversion of the bottom plate on the formation of spheroids, a pipette can be used to directly punch holes in the bottom plate made of polystyrene and inject droplets to form hanging drops for spheroid culture [32] (Figure 3).

#### 2.1.3. Rotating Flask

The above two types of methods are used to prepare spheroids under static culture conditions. The rotating flask method can also be used for generating spheroids. With this method, the flask itself is rotated to prevent cell sedimentation and the contact between cells is promoted by continuous stirring, thereby generating spheroids. The fluid movement generated by stirring can provide dynamic microenvironment and promote cell proliferation and differentiation. To prevent cell sedimentation, it is necessary to control the flow rate of the solution, but an excessive flow rate will damage the cells due to the greater fluid shear force, which will reduce the survival rate of the cells. To address this problem, a National Aeronautics and Space Administration’s high aspect ratio vessel (NASA HARV) bioreactor was made. The device rotates around the *x*-axis. During the entire culture process, the cells remain suspended under very low shear stress, which simulates the microgravity environment. Spheroids were successfully formed for a human mammary cell line (BT 20), prostate cancer cell line (PC3), and glioma cell (HBR 84) with this device [33].

#### 2.1.4. External Force

**Figure 4:**
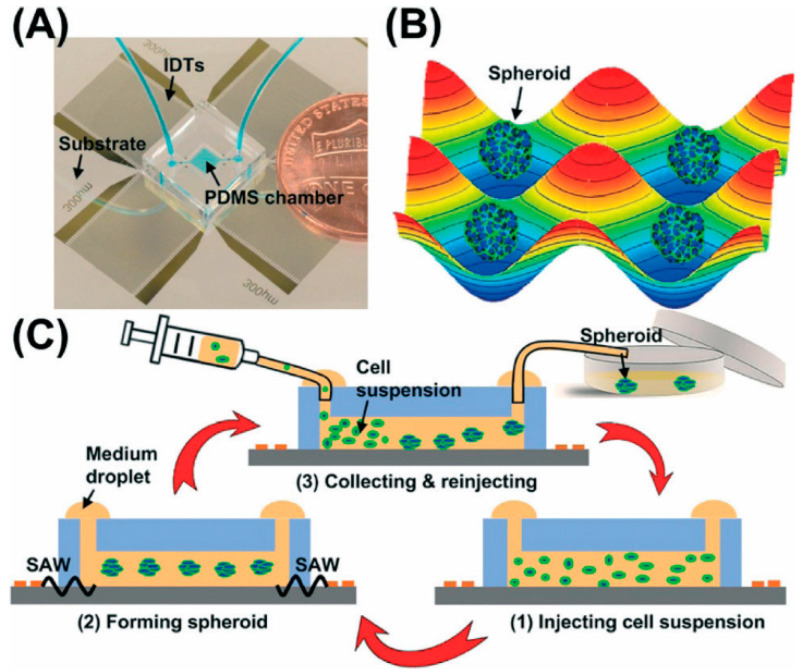
The concept of a 3D acoustic tweezers-based spheroid formation system. (**A**) The image of the PDMS chip coupled with interdigital transducers (IDTS). (**B**) The spheroids generated using the acoustic field. (**C**) The process of the whole spheroids formation based on 3D acoustic tweezers-based system. (Reproduced from Reference [39]).

To promote the aggregation of cells into spheroids, electric field [34], magnetic force [10,35,36], and sound waves can be used as external forces to accelerate cell aggregation. Ahadian et al. used dielectrophoresis to form three-dimensional embryonic stem cell aggregates in gel matrix hydrogels [37]. Souza et al. used a bio-assembler on magnetic levitation with gold-phage- magnetic iron oxide (MIO) suspension to prepare human glioblastoma spheroids through spatial control of the magnetic field [38]. Chen et al. used 3D acoustic tweezers to successfully prepare HepG2 spheroids of uniform size [39] (Figure 4). In their study, a surface acoustic wave (SAW) was used to generate cell spheroids. The Gor’kov potential field and microstreaming were produced by the SAWs. Cells were levitated by drag force from microstreaming in the vertical direction and aggregated via radiation force produced by the Gor’kov potential in the horizontal plane. Using this technique, more than 150 size-controllable spheroids were fabricated every 30 min. Although the external force method can directionally accelerate the aggregation of cells, it is difficult to evaluate the influence of external force on the physiological changes of cells. Moreover, the external force method usually requires professional equipment and operators, which undoubtedly adds difficulties to the preparation of spheroids.

### 2.2. Application of Biomaterials and Micromachining Technology in Preparation of Multicellular Spheroids

The above-mentioned traditional culture methods use the principle of spontaneous aggregation of a large number of cells and/or cell proliferation to form multicellular spheroids, but it can be difficult to use these methods to simulate cell–cell and cell–ECM interactions due to the very complicated microenvironment for cell growth [40]. Moreover, cell aggregation and/or cell proliferation can be affected by the synergistic effects of cell-binding sites, rigidity, hydrophilicity/hydrophobicity, electric charge, space limitation, and forces (such as gravity, centripetal force, centrifugal force, magnetic force, electric force and/or shear force) [41]. As traditional cultivation methods cannot provide a realistic cell microenvironment, perfectly biocompatible hydrogels as a scaffold material for simulating ECM have been used for generating MCSs in tissue engineering [42]. For example, Jeon et al. used a methacrylate alginate (OMA)/multi-arm polyethylene glycol (PEG) double-crosslinked hydrogel microwell system to prepare multicellular human adipose tissue-derived stem cell spheroids. By changing the size of the microwell, the size of the hydrogel microwell is controlled, and the biophysical and/or chemical properties of the hydrogel are modified locally to form a spatially controllable culture system [41]. Natural hydrogels have also been used for generating spheroids. Typical examples include collagen [44,45], chitosan [11,46], hyaluronic acid (HA) [47], and agar [48], and synthetic hydrogels represented by PDMS [49,50], PEG [51], and acrylic acid. The applications will be discussed later. Traditional culture methods such as hanging drop will impede the generation and subsequent analysis of spheroids due to the rapid consumption of nutrients and oxygen in the culture environment and the increase in metabolic waste and osmotic pressure. Another example is the rotation culture method. Although this method can generate spheroids at a high throughput, excessive shear will cause damage to the cells. Moreover, the traditional culture method usually uses static culture, which makes it impossible to continuously perfuse the culture medium and involves using a large amount of medium. The micromachining technology, for example, digital micromirror devices (DMDs), can be applied in the optical field to enable the use of the maskless lithography technology for generating spheroid culture devices. Microfluidic systems have emerged for years as an effective approach to the generation of MCSs. Compared to traditional methods, a microfluidic system can perfuse culture medium continuously, consume less reagent consumption, subject cells to an appropriate shearing force, and provide a dynamic environment for the formation of the spheroid. All these advantages are helpful in the generation of spheroids. Over the last decade of study, the idea of using hydrogels as scaffolds and using microfluidic systems to generate MCSs has been widely recognized in the industry, and has been successful in preparing HepG2 spheroids [52], human colon cancer cell (ATCC) spheroids [50], and normal human fibroblast (NHF) spheroids [53]. The research advances of these technologies will be examined in more detail in later sections.

#### 2.2.1. Hydrogel (Scaffold)

**Figure 5:**
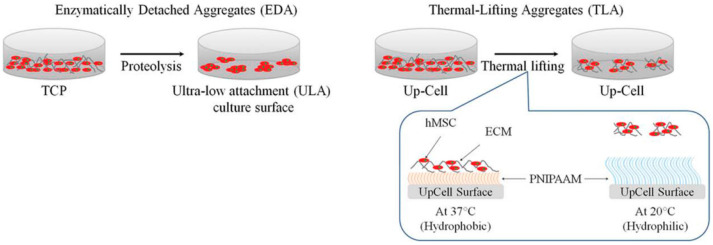
Schematics of human mesenchymal stem cells (hMSC) aggregate formation based on hydrogel. TCP: tissue culture plastics, PNIPAAM: poly-N-isopropyl acrylamide. (Reproduced from Reference [55]).

Hydrogel is rich in water and MCSs can be prepared in two ways: two-dimensional hydrogel surface and three-dimensional hydrogel embedding [54]. There are two main ideas to prepare spheroids at the two-dimensional level. The first is similar to the traditional low-adhesion surface. The hydrogel polymer is applied to the substrate. The polymer used has no obvious cell-binding sites [41], which cause the cells to be unable to attach to the substrate and promote the combination of cells and cells to form aggregates. The other idea is to find some thermo-sensitive hydrogels [55] (Figure 5), such as poly (*N*-isopropylacrylamide), at room temperature, so cells can grow on a substrate coated with polymer. When the cells are cultured into layers, the temperature of the bottom plates is changed, and the cell layer breaks away from the bottom plate, forming a cell sheet floating in the culture medium, and then the cell sheet spontaneously gathers to form a spheroid. It has been found that when cells are cultured on the bottom plate, the polymer can stimulate the cells to produce extracellular protein, which is very important for the later formation of spheroids [56]. The three-dimensional culture idea is to embed cells in a porous hydrogel, which acts as an extracellular matrix. The shape and size of the porous hydrogel pores can be controlled by computer technology, and the distance between the cells embedded in the hydrogel can be changed by controlling the internal gap [43]. By shortening the distance between cells, the cells are promoted to aggregate to form spheroids, and the porous structure allows aggregates to migrate in it, thereby promoting signal exchange between cells. It is found that the size of the spheroid in the hydrogel is associated with the initial cell density, the culture time, and the intrinsic properties of the hydrogel, especially the binding sites, hydrophilicity, and stiffness of cells [57]. An accurate description of cell stiffness is, however, not impossible via current laboratory research. It has been reported that cells are generally more likely to form polymers on soft hydrogels [58]. It has also been reported that hydrogels with higher hardness are conducive to the generation of spheroids [59]. Natural polymers have been used to form a scaffold for the generation of spheroids due to their excellent biocompatibility and biodegradability, and synthetic polymers have also been used to generate spheroids due to their better structural complexity. Next, we will summarize the applications of typical hydrogel materials.

##### Natural Polymers

Hyaluronic acid (HA) is originally isolated from the bovine vitreous body [60]. It is not sulfated or covalently bound to proteins, but exits in free form and non-covalent complex form. It is a glycosaminoglycan. HA has low viscosity to cells, which can promote cell–cell contact and facilitate the generation of spheroids [61]. HA can bind to receptors and proteins on the cell surface (such as CD44) to activate a variety of signaling pathways and regulate cell functions. In cancer cell research, HA binds to CD44 and affects tumor cell differentiation [62], proliferation, and metastasis. The production and metastasis of tumor cells are often accompanied by changes in the microenvironment. Studies have found that with the production of cancer cells, epithelial cells undergo epithelial mesenchyme, and the HA content in the microenvironment increases, achieving HA enrichment [63]. The enrichment of HA further promotes the proliferation of cancer cells. HA has been successfully made into hydrogels [64], scaffolds [65], fiber nets [66], and other structures to generate multicellular spheroids.

Collagen is the most abundant protein in mammals, and the most common type of collagen is collagen type I [67]. Collagen can be extracted directly from ECM. Collagen is an excellent cell attachment matrix. Cells can recognize and bind to collagen through integrin receptors [68]. In order to study the influence of the pore size of the collagen scaffold on cartilage regeneration, Zhang et al. prepared collagen scaffolds with four pore sizes: 150–250, 250–355, 355–425, and 455–500 μm. The results showed that 150–250 μm was the best choice for cartilage regeneration [59]. At present, collagen has been combined with microfluidic technology to rapidly produce collagen microspheroids while maintaining the viability of cultured cells [69]. Kaufman et al. found that when the cells cultured on the base gel are covered by collagen type I, the cells can be rearranged to form a spheroid [70]. Ma et al. embedded human glioma cells in collagen and formed hypoxia to simulate the tumor microenvironment in vitro. The results showed improved proliferation, spheroid formation and invasion of U87 glioma cells transfected with hypoxia-inducible factors (HIFs) compared to non-treated cells [45]. Kim et al. used collagen to culture human bladder cancer cell lines SBT31A and T24 that express cyclin D1b mRNA to study the ability of cyclin D1b siRNA to inhibit cancer. They found that the expression of cyclin D1b can be limited by inducing cell apoptosis, which inhibits cancer cell stemness and epithelial-mesenchymal transition and thereby inhibits the malignant phenotype of bladder cancer cells [44].

Agarose is a low-cost linear polymer with uniform gel structure and high water content; it features non-toxicity, good transparency and biocompatibility, and good permeability [71], which is conducive to the diffusion of oxygen and nutrients. These advantages make it suitable for generating MCSs [53,72,73]. Tumor spheroids can summarize the specificity of tumor better than two-dimensional cell culture, but most of the existing culture systems are only suitable for forming small-size spheroids. Tang et al. generated porous analytical agarose molding for the culture of U87-MG human glioblastoma, and successfully generated a U87-MG tumor spheroid with a diameter of 1.4 mm. They found that the size of the tumor spheroid did not increase with the density of the inoculated cells [48]. Tumor spheroids are used as an in vitro model for evaluating anticancer drug combination therapy. Barros et al. injected agarose into a micromold to form an agarose structure with spherical microwells and successfully cultivated PANC-1 spheroids using this structure. By changing the ratio of the therapeutic drug doxorubicin, i.e., resveratrol, the spheroids were tested for drugs, and then the therapeutic effect and synergy potential of the drugs were evaluated. They found that when a higher ratio of resveratrol was used, the viability of the cells was greatly reduced. Similar conclusions were validated in the two-dimensional cell model, showing considerable potential for the combined drug treatment method to deliver synergistic effects [74].

##### Synthetic Polymers

As a hydrophobic organic silicon material, poly-dimethylsiloxane (PDMS) has been widely used in the generation of MCSs due to its high transparency, non-toxicity, and good chemical stability and physiological inertia [75,76,77]. Shi et al. prepared PDMS concave microwells using photoresist SU-8 as a template and used them to generate chondrocyte spheroids under low oxygen conditions. They found that the combination of the spheroid model and hypoxic conditions significantly increased the expression of collagen II and aggrecan at protein and mRNA levels. HIF-1α can directly regulate the expression of aggrecan, while HIF-2α regulates the expression of Col2a2 and aggrecan primarily by regulating the Sox-9 gene [78]. To improve the volume of the generated spheroids and make them better simulate the size of real tumor tissues in vivo, Ratnayaka et al. generated HepG2 spheroids with a volume of 44 mm^3^ by combining the use of a suspension drop method and a PDMS well method [79]. Oxygen is a necessary substance during cell growth and reproduction. Anada et al. only used PDMS to prepare an oxygen-permeable chip to generate HepG2 spheroids to increase the concentration of oxygen in the culture environment. As a control, they used acrylic acid to prepare a non-oxygen-permeable chip for the spheroid culture. They found that the diameter of the spheroids on the oxygen-permeable chip increased significantly, the hypoxia and survival thresholds of the HepG2 spheroids cultured on it reached 400 and 600 mm, respectively, the cell growth was significantly enhanced, and anaerobic glycolysis was significantly reduced [80]. To overcome the shortcomings of the hanging drop method that the culture medium is not easy to replace and the spheroids are not easy to transfer, Kim et al. used a method in which a droplet array chip (DAC) containing the spheroids was contacted with a DAC to transfer the spheroids and replace the medium [81].

**Figure 6:**
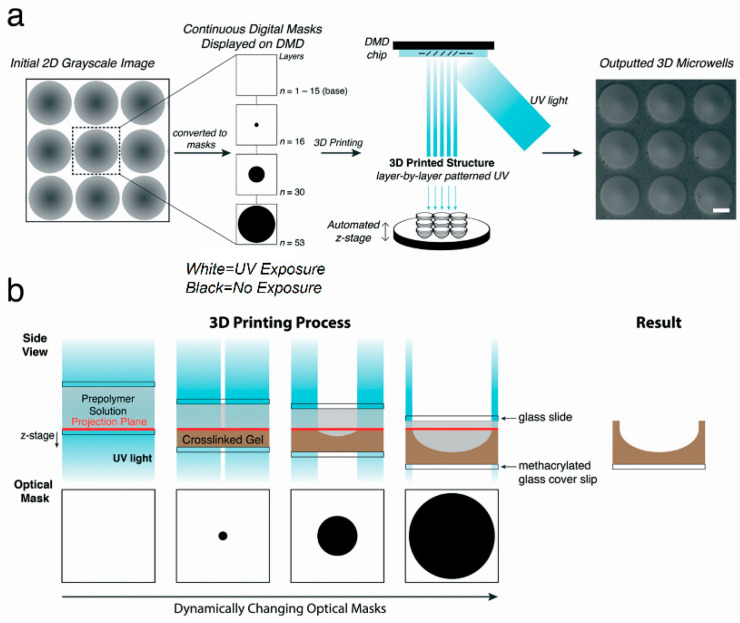
(**a**) Concave hydrogel microstructures for spheroid culture were fabricated using a light-based 3D printing process. (**b**) Cross-sectional schematic of the 3D printing process. All scale bars = 200 μm. (Reproduced from Reference [82]).

Poly-ethylene glycol (PEG) is characterized by good water solubility, non-toxicity, and good stability. It has also been widely used in the generation of spheroids [43]. Traditional photolithography technology requires a separate mask. Using ultraviolet light and a digital micromirror device (DMD), Hribar et al. performed nonlinear 3D projection to prepare concave hydrogel microstructures (Figure 6), and generated breast cancer and induced pluripotent stem cell spheroids. Through immunofluorescence and histochemical staining, they observed that the breast cancer spheroids cultured to the 10th day formed a hypoxic core, which was consistent with the in vivo tumor model. Both spheroids were successfully cultured for 10 days, verifying that long-term culture of spheroids can be achieved on this structure. The application of stereolithography technology can greatly improve the efficiency of microstructure preparation, and can enable precise control of microstructures [82]. The study found that the tumor microenvironment contained ECM components such as collagen and fibrinogen. To examine the role of fibrinogen in cancer progression, Pradhan et al. used a dual-photoinitiator, aqueous-oil emulsion technique to encapsulate MCF7 cells in PEG-fibrinogen hydrogel microspheroids. Using scanning electron microscopy and fluorescence imaging analysis, the apico-basal polarity of cells appeared to be drastically reduced in tumor microspheroids, to a level significantly lower than that of the spheroids formed by self-aggregation. This indicated the beginning of the epithelial-mesenchymal transition and the malignant transformation of the cells. As revealed by the ultrastructural analysis of the tumor globules and tumor microspheroid, the tumor microspheroids exhibited more disordered cell arrangement and smaller cell size. The MCF7 spheroids in the microspheroids showed higher Young’s modulus of 4700 ± 650 Pa, which could be attributed partly to the increase in the hardness of PEG-fibrinogen hydrogel [51].

There are some other synthetic polymers used in the generation of multicellular spheroids. For example, HepG2 spheroids have been successfully generated using poly(N-isopropylacrylamide) (PNIPAM), such as poly(2-hydroxyethyl methacrylate) (PHEMA) [52,83]. Zhao et al. generated NIH3T3 cell spheroids using hydrogel films with swelling-induced wrinkling patterns [84], and Rosellini et al. generated A-549, 293-T, KB, and MRC-5 spheroids using microplates coated with poly-2 hydroxyethyl-methacrylate (PHEMA) [85].

#### 2.2.2. Microfluidic Systems

Compared with non-microfluidic systems, microfluidic systems can realize continuous infusion of the culture medium while ensuring high cell activity. Moreover, they can be used for drug testing, live/dead cell staining, and other characterization analysis on microfluidic chips. A microfluidic system can reduce the use of cells and the consumption of reagents and is suitable for high-throughput screening. Owing to their combination with hydrogels, microfluidic systems have been able to achieve the high-throughput generation of uniform-sized spheroids in the generation of MCSs, and are showing a tendency to reduce labor intensity and automatic monitoring. Now, microfluidic systems have become an important tool in the field of biomedicine. Depending on whether spheroids can be formed, microfluidic chips can be divided into microfluidic spheroid formation chips and microfluidic spheroid culture chips. Various microfluidic spheroid culture chips have been fabricated. They are divided into two categories according to their formation processes: the formation of spheroids based on emulsion, and the formation of spheroids based on microwells or U-shaped microstructures.

##### Emulsion Technology

**Figure 7:**
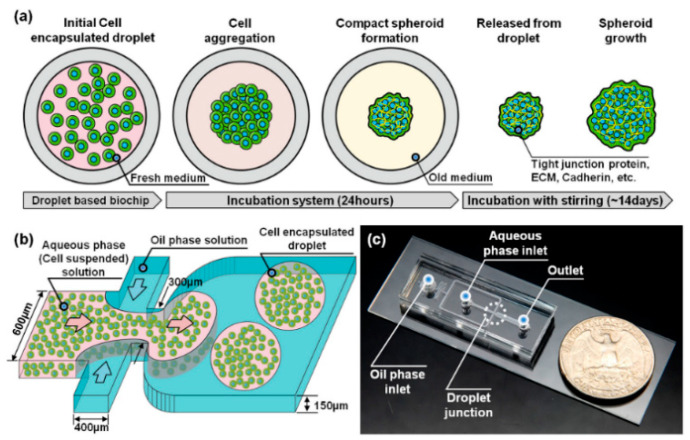
(**a**) The formation of a 3D tumor spheroid model from a single cell in micro-droplet. (**b**) A droplet-based microfluidic system for 3D tumor spheroid model generation. (**c**) The optical image of the whole device. (Reproduced from Reference [91]).

The incompatible solution is injected through the microfluidic channels of a microfluidic chip. The solution in one channel contains cells. When the solution of each microfluidic channel meets in the intersection area, the solution containing cells will form emulsion droplets due to the shear force of the fluid in the other solution. Cells are trapped in the emulsion droplets to form a closed microenvironment, and then form spheroids through the aggregation of cells. At present, single [3,69,86,87], two [88,89], and three [90] layers of emulsion droplets have been developed. With this method, emulsion droplets can be prepared with high throughput and reduced labor intensity. Kwak et al. used emulsion technology to generate human breast cell spheroids, and the droplet generation speed reached 1000 drops/min [91] (Figure 7). The hydrogel mentioned above has been widely used in emulsion technology to form emulsion droplets because of its good biocompatibility. External stimulation is required for hydrogels in emulsion droplets to achieve gelation. For example, thermal responsive hydrogels need to be solidified by changing the temperature. UV irradiation is required for photosensitive hydrogels to induce light crosslinking. Alginate can be prepared by injecting ionic solutions [92]. Chen et al. prepared an alginate-based microfluidic system, generated breast tumor cell spheroids, and tested them with doxorubicin. They found that as the concentration of doxorubicin increased, the cell’s activity and proliferation ability decreased significantly [42]. However, the disadvantage of emulsion technology is that after the emulsion droplets are formed, the separated droplets restrict the cells from acquiring nutrients, which may lead to cell necrosis. To solve this problem, leveraging the high throughput of multi-phase microfluidics and ease of perfusion with single-phase microfluidic technology, McMillan et al. prepared a new microfluidic platform that can be used to prepare and culture spheroids in culture medium and in alginate gel scaffolds [93]. In general, emulsion technology has been widely recognized for the speed of droplet preparation, and the formed spheroids can be used for in vitro drug screening.

##### Microwell and U-Shaped Microfluidic System

**Figure 8:**
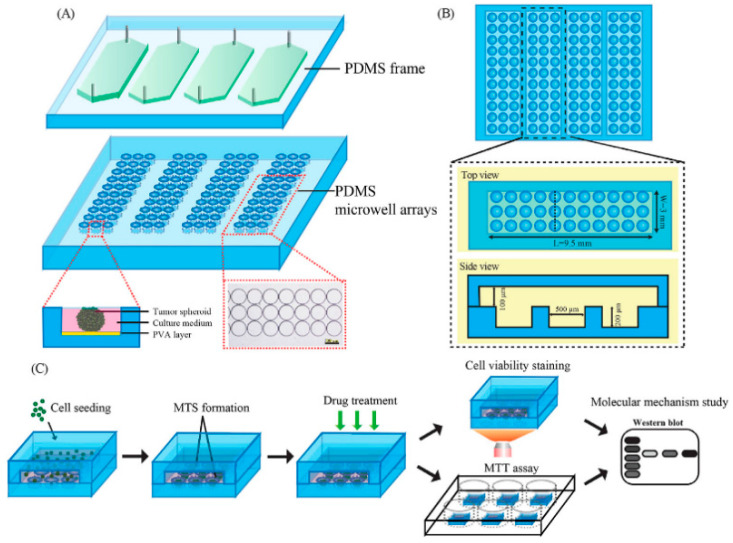
(**A**) Schematic illustration of the microfluidic device for MTS formation and drug screening application. (**B**) Top and side views of the microfluidic device. (**C**) The process of MTS formation and drug screening. (Reproduced from Reference [6]).

The generation of spheroids can also be achieved by using microfluidic chips with microwells [6,23,94,95] (Figure 8) or U-shaped microstructures [12,96,97]. The basic working principle of this method is that a solution containing cells is injected into the chip through a microchannel, and the cells are captured by microwells or U-shaped microstructures. The cells aggregate to form spheroids, and the medium can be continuously injected into the chip through the microchannel, continuously providing nutrients to the cells and taking away the waste produced by cell metabolism. This type of microfluidic chip can be used for long-term culture of spheroids, and thereby it can find applications in vitro cancer research and drug detection, which will be covered later in this article. Reducing cell loss during spheroid generation and controlling the formed spheroids in microwells or U-shaped structures is an important indicator for evaluating the design of microfluidic chips [98,99]. Fu et al. used in-situ photolithography to prepare a microfluidic chip with a U-shaped microstructure to generate MCSs. The influence of the angle on the cell capture efficiency was studied by changing the tilt angle of the chip (0, 45 and 90 degrees, respectively). They found that the cell capture efficiency was highest when the chip was tilted at 90 degrees [96]. Microfluidic chips can not only use a single cell to generate homogeneous spheroids, but also use a variety of cells to generate heterogeneous spheroids to explore the interaction between different types of cells in the process of spheroids formation [95,99,100]. Zuchowska et al. co-cultured breast cancer cell line MCF-7 and human mammary fibroblasts (HMFs) to generate spheroids, and generated MCF-7 spheroids as a control to evaluate the therapeutic effect of photodynamic therapy. Studies have found that the content of nano-TPP and ROS is higher in the homogeneous spheroids, which indicate that the heterogeneous spheroids are much more resistant to photodynamic therapy [95].

## 3. Applications of MCSs

### 3.1. Tumor Research

**Figure 9:**
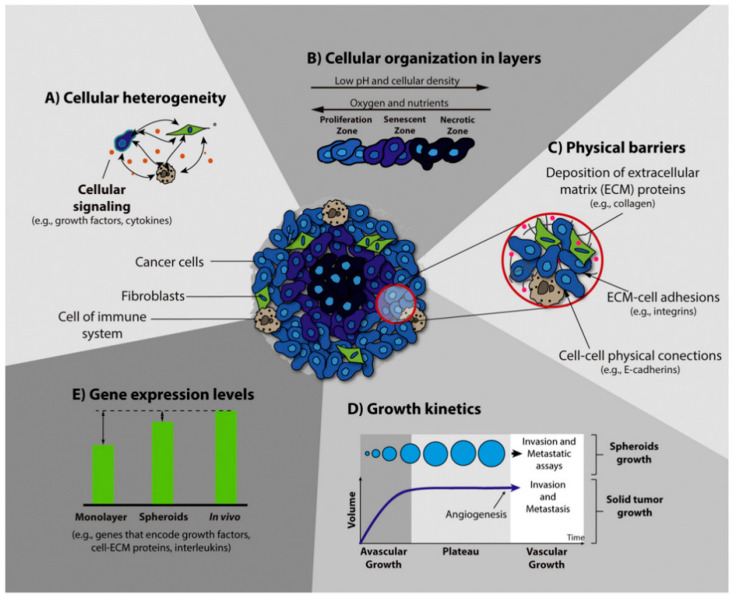
Schematic representation of the main characteristics of 3D spheroids that are crucial for their application in the screening of anticancer therapy. (Reproduced from Reference [17]).

Three-dimension models for tumor research in vitro include multicellular layers, matrix-embedded culture, hollow fiber bioreactor, ex vivo cultures, and MCSs [101]. MCSs have become an important model for cancer research because they can co-culture a variety of cells to form heterogeneous spheroids and can mimic cell–cell and cell–ECM interactions [12,17,102,103,104]. Tumor cells were co-cultured with fibroblasts and endothelial cells to simulate the complex cell microenvironment in vivo [36,105,106]. A schematic representation of the main characteristics of 3D spheroids is shown in Figure 9. MCSs can simulate the characteristics of angiogenesis, invasion, and metastasis. Generally speaking, angiogenesis represents the pathological changes of tissues. Chiew et al. co-cultured HepG2 hepatocellular carcinoma (HCC) cells and endothelia cells and found that endothelial cells form tubular networks, and endothelial cells can enhance differentiation under the action of angiogenic factors [105]. Similarly, Esendagli et al. co-cultured mouse breast cancer cells, fibroblasts, and macrophages to study the effects of the other two on breast cancer cells. The study found that the number of spheroids formed by co-culturing fibroblasts and breast cancer cells was lower than that of breast cancer spheroids prepared alone, and when macrophages were added, the frequency of spheroids increased. These results indicate that fibroblasts have a negative effect on the formation of breast cancer spheroids [16]. At present, researchers have found that epithelial-mesenchymal transition (EMT) is a key stage in the development of cancer, with decreased cell adhesion and increased tumor metastasis and invasiveness [45,106,107]. Malignant glioma is the most aggressive type of brain tumor. Hypoxia is an independent prognostic factor. Ma et al. created a hypoxic microenvironment for the spheroid of U87 cells by using hypoxia-inducible factor (HIF). After 10 days of culture, it was found that the spheroids transfected with HIF had stronger invasiveness, and the anterior invasive cells took on a mesenchyme-like shape [45]. When the diameter of multicellular tumor spheroids (MCTSs) cultured in vitro exceeds a certain limit, molecular diffusion gradients will be formed inside the MCTSs, resulting in the inability of the internal cells to obtain enough nutrients and the accumulation of metabolic wastes. The spheroids will form a typical three-layer structure, comprising an internal necrosis area, an intermediate static area, and an external proliferation area [105]. The diffusion range of oxygen and other biomolecules is about a few hundred microns, so an MCS of 500 μm is an ideal model for cancer research [108].

### 3.2. Drug Screening

**Figure 10:**
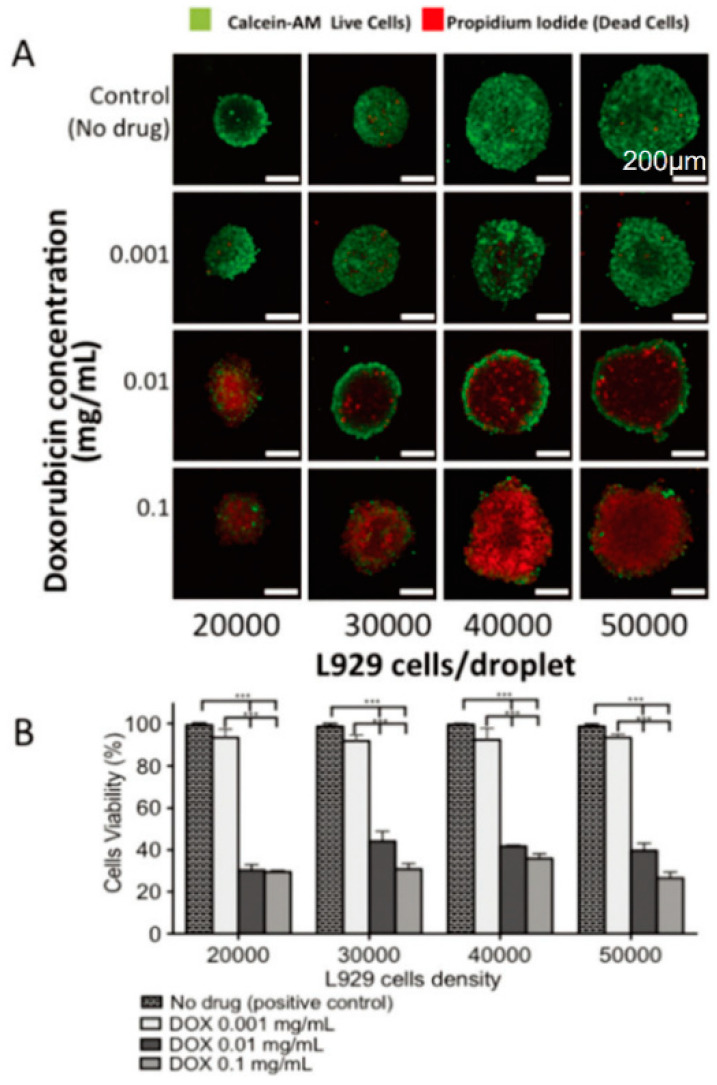
(**A**) After being treated with different concentrations of doxorubicin, fluorescence images of the L929 cell spheroids with different densities were obtained using confocal microscopy. (**B**) Cell viability in the 3D spheroids using different cell densities and concentrations of doxorubicin, obtained using imageJ analysis. (Reproduced from Reference [29]).

Drug screening in vitro is needed before drug clinical trials. Animal models are recognized as the most ideal in vitro models. However, using animal models for drug screening involves high costs and is complicated by moral issues. Traditionally, a two-dimensional cultured cell model is required before animal experiments. However, due to the absence of cell–cell and cell–ECM interactions in the culture process, this model cannot really simulate the real situation of the human body, which leads to poor drug efficacy in clinical trials. The MCSs model has been used for in vitro drug screening and has also been used for anticancer drug screening [6,29,109,110]. The liver plays an important role as an organ of drug metabolism in vivo. To fix the position of the spheroid and limit the size of the spheroid, Xia et al. prepared tethered spheroids and verified that CYP450 could be produced by induction [110]. Drugs such as doxorubicin [42], cisplatin [8,111], and paclitaxel [6,108] have been used in drug resistance and sensitivity experiments on spheroids. For example, doxorubicin, an anthracycyline drug, has been widely used. The test of doxorubicin by MCSs revealed that as the concentration of doxorubicin increases, the cell viability decreases, and MCSs have stronger drug resistance compared with two-dimensional culture (Figure 10). This demonstrates that MCSs can be used as a platform for drug screening [29].

### 3.3. Tissue Engineering

**Figure 11:**
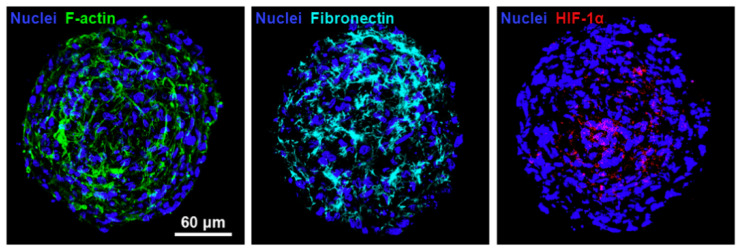
Immunofluorescence images of human umbilical vein endothelial cells (HUVEC)/cbMSC cell spheroids stained with antibodies against F-actin, fibronectin, and hypoxiainducible factors(HIF-1α). The results indicate that cell spheroids possessed the ECM molecule fibronectin and hypoxiaresponsive transcription factor HIF-1α. (Reproduced from Reference [112]).

Tissue engineering is used to construct biological substitutes in vitro and inject them into the body to replace tissues and organs to achieve minimal damage to the human body [112]. Because of their good biocompatibility and biodegradability, hydrogels have been widely used in tissue engineering to form scaffolds. Huang et al. constructed hypoxia human umbilical vein endothelial cells/cord-blood mesenchymal stem cell spheroids and implanted them into a mouse ischemic hindlimb model to promote blood vessel formation and prevent tissue degradation (Figure 11) [112]. Mesenchymal stem cells have been used to achieve cartilage regeneration because of their self-renewal and multi-phase differentiation capabilities [113]. Hus et al. induced differentiation of human gingival fibroblasts (HGF) on chitosan, and found that HGF cells formed spheroids, and C×43 activity increased, which increased the cartilage differentiation potential of HGF [15]. Kim et al. co-cultured endothelia cells and stem cells to achieve high production of core-shell spheroids with controllable size. The spheroids can also rapidly induce vascular networks in micro-tissues [9].

### 3.4. Tumor-Immune-Cell Interactions

The anti-tumor activity of immune cells is mainly influenced by the complex cellular networks within tumors and tumor immune escape mechanisms [114,115,116]. T cells and Natural Killer (NK) cells are major effectors of antitumor immune responses. Multicellular tumor spheroids (MCTS) are recognized as relevant models to human pathologies for studying cancer immunotherapies. Courau et al. cultured human colon tumor-derived spheroids with allogeneic T and NK cells to assess the function [117]. The results indicated that these immune cells rapidly infiltrated cell line-derived spheroids, inducing immune mediated tumor cell apoptosis and spheroid destruction. Giannattasio et al. investigate infiltration and the cytotoxicity of NK cells using human cervical carcinoma cell spheroids and discovered that the destruction of a three-dimensional tumor spheroid took much longer when compared to the monolayer cultures when treated by primary human NK cells [118]. Similar work was also done by M. Lanuza and his coworkers [119]. They analyzed the efficiency of allogeneic NK cells on colorectal (CRC) human cell spheroid. The result indicated that colorectal tumour cell spheroids, which favoured the expression of the inhibitory immune checkpoint PD-L1, were killed by activated NK cells efficiently.

## 4. Challenges and Prospects

The application of MSCs in the biomedical field has built a bridge between two-dimensional cell culture models in vitro and the real environment in vivo. Traditional methods such as suspension drop and non-adhesive surface liquid covering culture have stood the test of time. The external fore-based method requires professional equipment and has an unknown impact on cells. At present, the MSCs technology is relatively mature and has been widely used in the preparation of spheroids. Recent advances in this technology include new microfluidics systems and new hydrogels, which are among the most effective ways to simulate the microenvironment in the body. Hydrogels play an important role in simulating ECM. Microfluidics allow for continuous infusion of nutrients and thus long-term culture of spheroids. However, biomaterials and microfluidic technology are not only higher in cost but also hard to operate. These limitations hinder the wider application of MCS generating techniques. The questions of how to simply and rapidly generate microfluidic chips and how to conveniently connect with analytical instruments for high-throughput analysis will be the focuses of future research. Future advances in technologies such as microlens, atomic force microscopy, and 3D printing are expected to allow for more precise control of the microenvironment when generating MCSs.

For 3D MCS culturing, although MCSs can be generated successfully by various methods, the diameter of spheroids that can be formed is still limited to 200–400 μm due to molecular diffusion; when the diameter of spheroids is enlarged again, there will be necrosis areas in the middle of the spheroids due to the lack of nutrients and oxygen and the accumulation of metabolic wastes. This in turn limits the further development of tumor spheroids in tissue engineering. Generating heterogeneous spheroids to form micro-tissues and induce angiogenesis can be a promising method to expand the diameter of tumor spheroids in the future.

Although hydrogels play a part in simulating ECM, the cell–cell and cell–ECM signal transduction is not completely clear, and the mechanical, structural, and adhesive properties of hydrogels will impair the generation of spheroids. Different experimental results may occur due to different properties of the same material. In the future, it is necessary to develop standard experimental schemes to ensure the reproductivity of experiments.

In short, high throughput, convenience, and speed are required for the generation of spheroids. With a better understanding of the mechanism of signal transmission and the microenvironment between cells, more accurate 3D MCSs models will be generated.

## 5. Conclusions

Cell spheroids, as one of the most typical models of 3D cell culture, allow cells to establish cell–cell and cell–extracellular matrix connections to form a specific 3D structure that better simulates the complex intracellular microenvironment in vivo. In this review, methods for MCSs generation and their respective advantages and disadvantages were summarized, and the advances of hydrogel and microfluidic systems in the generation of spheroids were highlighted. Then, various applications of MCSs in cancer research and other aspects are presented and the current limitation, challenges, and the development direction are summarized.

## Figures and Tables

**Table 1:** A comparison of MCS generation techniques.

Generation Methods			Advantages	Disadvantages	References
Traditional generation methods	Non-adhesive surface liquid covering (the microwell arrays method)		Easy to operate Low sheer stresses High yield Low cost	Labor intensive Variation in MCSs size and shape Inability to stimulate cell-ECM interactions	[7,21,22,23,24,25,26,27,28]
	Hanging drop		Easy to operate Good size control Low sheer stresses Co-cultivation of multiple cells	Labor intensive Low yield Difficulties in mass production Difficult to change the medium Difficult to transfer the spheroid	[29,30,31,32]
	Rotating flask		Mass generation Easy to operate Long-term culture Dynamic microenvironment Co-cultivation of multiple cells	High sheer stresses Variation in MCSs size and shape Inconvenient to observe the generation process of the spheroid Inability to stimulate cell-ECM interactions	[33]
	External force		Rapid generation Good size control Co-cultivation of multiple cells	Requiring professional equipment The potential impact of external forces on cells is unknown	[10,34,35,36,37,38,39]
Biomaterials (scaffolds) and microfluidic technology	Hydrogel (scaffold, cell sheets)	Natural polymers	Realistic microenvironment High yield Good size control Low sheer stresses Labor saving Aggregates of different shapes can be generated Co-cultivation of multiple cells	Requiring professional equipment Higher requirements for operation Higher cost	[40,41,42,43,44,45,46,47,48,49,50,51,52,53,54,55,56,57,58,59,60,61,62,63,64,65,66,67,68,69,70,71,72,73,74]
		Synthetic polymers			[75,76,77,78,79,80,81,82,83,84,85]
	Microfluidic	Emulsion technology	Realistic microenvironment High yield Long-term culture Good size control Low sheer stresses High-throughput analysis Labor saving Dynamic microenvironment Generate aggregates of different shapes Co-cultivation of multiple cells Low reagent consumptionLow cell usage	Requiring professional equipment Higher requirements for operation Higher cost	[3,86,87,88,89,90,91,92,93]
		Microwell and U-shaped microfluidic system			[6,12,23,94,95,96,97,98,99,100]
